# Commentary: Incorporating concepts and methods from causal inference into life course epidemiology

**DOI:** 10.1093/ije/dyw103

**Published:** 2016-10-06

**Authors:** Bianca L De Stavola, Rhian M Daniel

**Affiliations:** LSHTM Centre for Statistical Methodology and Department of Medical Statistics, London School of Hygiene and Tropical Medicine, London, UK

## Introduction

The review by Ben-Shlomo *et al.*^1^ highlights how life course epidemiology is evolving and adapting to accommodate increasing access to data on novel dimensions and over extended periods. This enriched framework raises ever greater methodological challenges, leaving statisticians like us daunted by the task of translating life course enquiries into suitable analyses of the data at hand.

Take for example Figure 4 of Ben-Shlomo *et al.*.^1^ This is very useful for gaining a ‘big picture’ understanding of a complex area such as ageing, and for establishing which processes may benefit from a more detailed investigation. However, the leap from such a diagram to a specific data analysis should not be (and is not typically) made without greater thought. We will argue in this commentary that some recent developments from the field of modern causal inference may be helpful in this regard. First, in order to state unambiguously the question (or questions) of interest, the potential outcomes framework, a cornerstone of modern causal inference thinking, is invaluable. Then, the conceptual framework should be refined to a causal directed acyclic graph (DAG) relevant to the question, and the causal DAG should be formally interrogated to see if the question can be addressed, and if so how. Indeed, depending on the question, the causal DAG and the data available, we may find that standard statistical methods traditionally used in epidemiology are sufficient; in other settings we may find that more novel techniques are needed.

We will discuss each of these points next, mentioning also the issues of missing data and measurement error, as well as highlighting concerns about the difference between the processes which are the focus of investigations and their manifestations in observed data.

## What is the question of interest?

For illustration, we take an example briefly discussed by Ben-Shlomo *et al.*,^1^ namely the relationship between nutrition and type II diabetes. Whereas the broad aim of a project may be to understand the effect of nutrition across the life course on the risk of developing adult type II diabetes, a more specific question must be established before we can proceed. In this section, we will highlight the range of different questions that may be of interest, and how they can be unambiguously distinguished using the potential outcomes notation.

### Potential outcomes

Suppose for simplicity that nutrition is reliably measured (e.g. via detailed food frequency questionnaires together with analysis of urinary samples) twice during the life course on a cohort of people: once in childhood and again in early adulthood. Let $X_{1}$ and $X_{2}$ denote relevant summaries of nutritional status in childhood and early adulthood, respectively, and let the binary variable Y denote the development of type II diabetes by age 70 years, say. Let $Y(x_{1})$ be the potential outcome, i.e. the value that Y would take if we were hypothetically to intervene on $X_{1}$ and set it to the value $x_{1}$. We can similarly define potential outcomes $Y(x_{1})$ for hypothetical interventions on $X_{2}$. We may also define $Y(x_{1},x_{2})$, the value that Y would take were we to intervene on both $X_{1}$ and $X_{2}$ and set them to $x_{1}$ and $x_{2}$, respectively. We will now use this very simple example to illustrate how subtly different questions of interest can be articulated using these potential outcomes.

### Total and joint effects

The total causal effect (TCE) of $X_{1}$ on Y (including both its direct effect and its indirect effect via $X_{2}$) can be expressed as a comparison of the distribution of $Y(x_{1})$ for different values of $x_{1}$. Often the mean is compared, as in ${TCE}_{1}(x_{1})=E\{Y{(x}_{1})\}-E\{Y(x_{1}^{*})\}$, where $x_{1}^{*}$ is a reference (or baseline) value of $X_{1}$; for binary $X_{1}$, ${TCE}_{1}=E\{Y\left(1\right)\}-E\{Y\left(0\right)\}$. If Y were a time-to-event outcome, such as time to onset of type II diabetes, we could compare the survivor functions of the potential outcomes, for example.^2^ The TCE of $X_{1}$ would be of public health interest, for example if primary school nutrition programmes were being considered. If a public health nutrition initiative targeted at adults would instead be considered, then the causal effect of $X_{2}$ on Y, ${TCE}_{2}$ would more likely be of interest, which would involve a comparison of the distribution, e.g. the mean, of $Y(x_{2})$ for different values of $x_{2}$. Alternatively, the likely impact of a general intervention such as increased taxation of unhealthy food would more naturally lead to a comparison of the distribution of $Y(x_{1},x_{2})$ for different values of $x_{1}$ and $x_{2}$: this is the joint effect of $\left(X_{1},X_{2}\right)$ on Y.^3^^,^^4^

### Controlled direct effects and conceptual models in life course epidemiology

Another possible aim might be to gain a better biological understanding of the timing and strength of the mechanisms linking nutrition to type II diabetes prevalence. In this case we might compare the distribution of $Y(x_{1},x_{2})$ for different values of $x_{1}$ but for a fixed value of $x_{2}$, known as a controlled direct effect, for example ${CDE}_{1}(x_{2})=E\{Y\left(x_{1},x_{2}\right)\}-E\{Y(x_{1}^{*},x_{2})\}$.^5^ Evidence of variation in ${CDE}_{1}(x_{2})$ for different values of $x_{2}$ would indicate that the effect of nutrition in childhood on the risk of diabetes varies according to the level at which adult nutrition is set. This would support the so-called pathways model discussed in life course epidemiology^6^^,^^7^ according to which sensitive periods of exposure interact in their impact on risk (see below for further discussion of this). In the absence of such effect heterogeneity, we could compare the common ${CDE}_{1}$, which represents the effect of $X_{1}\;$that is not mediated by $X_{2}$, with the total causal effect of $X_{2}$, ${TCE}_{2}$. A similarity between them would support the cumulative exposure model*.* This is because the similarity (in addition to the lack of interaction) implies that experiencing the exposure during each of these two periods (directly) influences the risk of type 2 diabetes by the same amount, and hence it is the cumulative exposure, rather than the timing of it, that matters. If instead one or other effect were much smaller than the other, there would be support for the sensitive period model, and one or other effect being zero would support the critical period model.^6^^,^^8^

### Interaction versus effect modification

Returning again to the pathways model, there are two subtly different possible questions even here, which can clearly be articulated using potential outcomes, namely the difference between interaction and effect modification.^9^ For ease of explanation, suppose that $X_{1}$ and $X_{2}$ are both binary, and that we are interested in comparing the means of the potential outcomes. An interaction is said to be present if the two CDEs differ, i.e. if E{Y(1,1)}-E{Y(0,1)}≠E{Y(1,0)}-E{Y(0,0)}. This is the same as saying that: E{Y(1,1)}-E{Y(1,0)}≠E{Y(0,1)}-E{Y(0,0)}, i.e. the causal effect of changing adult nutrition on the risk of type II diabetes differs according to the level at which we set childhood nutrition. Conversely effect modification, as defined by VanderWeele,^9^ would be present if the causal effect of changing adult nutrition on the risk of type II diabetes differs between those who in reality have different childhood nutrition statuses, i.e. if $E\{Y(x_{2}=1)-Y(x_{2}=0)|X_{1}=1\}\neq E\{Y(x_{2}=1)-Y(x_{2}=0)|X_{1}=0\}$. (We explicitly write $Y(x_{2}=1)$ instead of Y(1) here, to clarify that the hypothetical intervention being considered is on $X_{2}$ rather than $X_{1}$.)

In this literature, interaction has a causal connotation with respect to both exposures, whereas effect modification is causal only with respect to (in this case) the later exposure $X_{2}$. Which of these questions is of interest will depend on the broader aim of the investigation, and will have an impact on how the data are analysed.

### Effect decomposition

Alternatively, we might be interested in effect decomposition, i.e. in asking what proportion of the effect of childhood nutrition on type II diabetes is mediated by early-adult nutrition. For these questions, so-called natural direct and indirect effects are relevant,^10^^,^^11^ and can again be unambiguously stated as a counterfactual comparison; the natural direct effect, for example, is a comparison of the distribution of $Y\{x_{1},X_{2}(x_{1}^{*})\}$ for different values of $x_{1}$ where $X_{2}(x_{1}^{*})$ is the potential value of $X_{2}$ were we to set $X_{1}$ to $x_{1}^{*}$. More specifically, the natural effect of $X_{1}$, expressed as a mean difference, is defined as ${NDE}_{1}=E\{Y\{x_{1},X_{2}\left(x_{1}^{*})\}\right\}-E\{Y\{x_{1}^{*},X_{2}{(x}_{1}^{*})\}\}$. These effects and their estimation have received much attention in the recent causal inference literature.^5^ Since these effects involve nested counterfactuals, they require very strong untestable assumptions for identification, assumptions that could not even be hypothetically verified in an experimental setting. For this reason, attention is currently being diverted to more policy-relevant effects known as interventional direct and indirect effects identifiable under weaker conditions.^12^ We expect that these effects will soon be estimated in applications in life course epidemiology.

### Multiple exposures/mediators

Suppose we had an additional exposure time point, such as nutrition during infancy; we would then be in a setting in which we could potentially be interested in the joint effects of more than two exposures,^3^^,^^4^ or we might be interested in effect decomposition with multiple mediators.^13^^,^^14^

### Comment

In any particular study, it is unlikely that all of the above would be relevant. Our main message, however, is that life course investigations are causal enquiries. Familiarity with the modern causal inference literature—and with the subtly different flavours of causal effects defined therein—has the potential to aid researchers in formulating and communicating the question(s) of interest.

## What is the appropriate causal DAG and what can it tell us?

Once the question of interest has been stated, establishing whether it can be answered under plausible assumptions using the data at hand, and if so how, can be aided by drawing a causal diagram [more precisely, a causal directed acyclic graph (DAG)].^15^ Such a causal DAG should reflect a priori subject-matter knowledge regarding the likely causal structure of the variables being studied. Unlike conceptual frameworks, causal DAGs are well-defined mathematical objects that can be interrogated using procedures such as d-separation;^16^ as such, care must be taken to draw them correctly, otherwise the resulting conclusions will be unreliable. Further, although a unique causal DAG can never be determined from the data and hence subject-matter knowledge is crucial, some candidate causal DAGs may be incompatible with the data, and thus compatibility should be investigated. There exists some recent technical literature on the various possible causal interpretations of DAGs,^17^^,^^18^ but one shared feature is that to be causal, any common (measured or unmeasured) cause of two or more variables in the diagram must itself be in the diagram.

**Figure 1.**
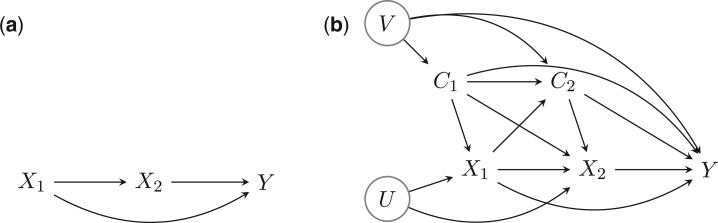
(a) A naïve causal DAG. (b) A more realistic causal DAG, with the unmeasured variables circled.

See, for example, our Figure 1 for the nutrition-diabetes example. The most naive causal DAG would assume no common causes of any pair of $X_{1},X_{2},Y$ and, under this assumption, finding a causally interpretable statistical analysis of the data would be straight-forward. More realistically, however, the nutrition-diabetes relationship will be confounded by a number of factors that we denote by C [e.g. socioeconomic position, physical activity and body mass index (BMI)]. In most life course settings, many of these confounders themselves will change over time (hence $C_{1}$ and $C_{2}$ in the figure), and to make progress, reliable repeated measures on these confounders would be needed. In particular, note that we have allowed the later confounders $C_{2}\;$to be affected by the earlier exposure $X_{1}$ (as would be expected, particularly with say physical activity and BMI). This dependence of $C_{2}$ on $X_{1}$ introduces a potentially problematic feature common in life course studies, namely time-dependent (or intermediate) confounding.

If Figure 1(b) were correct, standard regression methods could be used to make inferences about the total effect of $X_{2}$ (by adjusting for $X_{1},C_{1},C_{2}$), and also about effect modification by $X_{1}$ of the total effect of $X_{2}$ on Y.^19^ In the unlikely event that we could additionally assume no unmeasured common causes U of $X_{1}$ and $X_{2}$, then inference about the total effect of $X_{1}$ could also be made using standard methods upon adjustment for $C_{1}$. If, however, we wished to make inference about the joint effects of $X_{1},X_{2}$, or the controlled direct effect of $X_{1}$ on Y fixing $X_{2}$ to a particular value, or to learn about interaction between $X_{1}$ and $X_{2}$ in their effect on Y, then so-called g-methods that deal with time-dependent confounding would be needed.^3^^,^^4^ Effect decomposition using natural direct and indirect effects would not be possible in the presence of U, and would anyway require additional strong parametric assumptions (due to the presence of intermediate confounding).^20^^,^^21^ However, progress could be possible using interventional mediation effects^12^ using the observed (rather than counterfactual) distribution of $X_{2}$ given $X_{1}$ and $C_{1}$; see a related discussion by VanderWeele and Robinson.^22^

**Figure 2.**
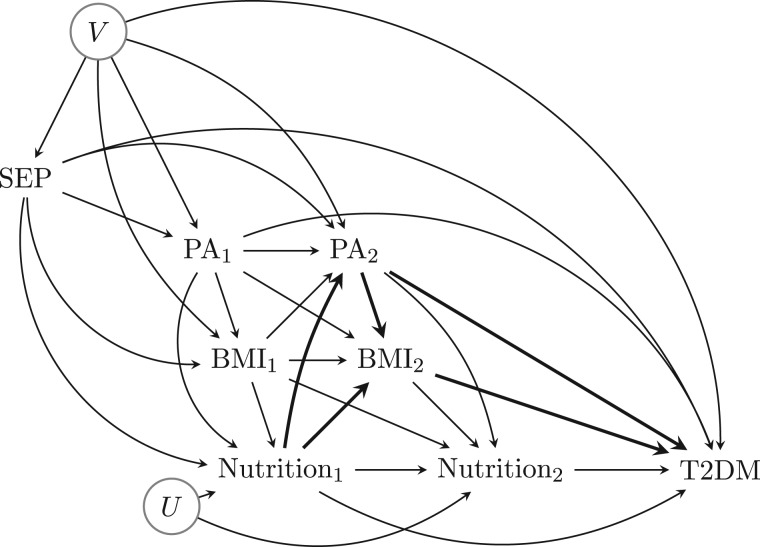
A causal DAG for the nutrition-diabetes example.

Figure 2 is an expanded version of Figure 1(b) specific to the nutrition-diabetes example. It highlights how controlling for adult physical activity and BMI ($C_{2})\;$would remove part of the effect of childhood nutrition, the part that they mediate (thicker arrows in the DAG), whereas not controlling for adult physical activity and BMI would confound the effect of adult nutrition. Hence a traditional regression approach would not achieve the estimation of the joint effects of childhood and adult nutrition (including their interaction), nor of the controlled direct effect of childhood nutrition.

## Missing data and measurement error

As well as confounding, other challenges facing those attempting to estimate causal effects in life course studies are: the likely depletion of participation during the course of follow-up, with the resulting missing data; and measurement error in particular when it affects a mediator in the considered analysis. The first challenge is a particularly relevant problem in studies of ageing, as also discussed by Ben-Shlomo *et al.*,^1^ where data missing due to death or other competing events present an especially difficult problem.^23^ Measurement error is ubiquitous in observational epidemiology and is arguably of even greater concern when it affects a mediator, with induced biases in opposite directions for direct and indirect effects.^24^^,^^25^ Inevitably when faced with incomplete and/or mismeasured data, as with confounding, assumptions have to be made and then sensitivity to these assumptions assessed. Causal diagrams present an opportunity to represent these assumptions, for example by including missingness indicators (see for example Daniel *et al.*^26^ and the extensions by Mohan, Pearl and others)^27^ or measurement error mechanisms in the DAG.^28^ The implications of different assumptions can then be formally assessed, and appropriate analyses, if they exist, identified.

## Processes versus snapshots

We end our commentary by drawing attention to a recent important cautionary note on the use of causal DAGs, by Aalen *et al.*,^29^ particularly relevant to studies in life course epidemiology. Conceptual frameworks, e.g. Figure 4 in reference 1, rightly concern processes, usually latent, such as the continuous-time evolution of an individual’s reproductive function or nutritional trajectory over the life course. However, when we draw causal DAGs that are to be useful for informing data analysis, we naturally focus on the ‘snapshot’ observations of this latent process that are available in our data. As long as the translation from process to snapshot is done knowingly and carefully, this is wise; however, if we treat the snapshots in our causal DAGs as if they actually represented the whole process, then mistakes can be made and conclusions adversely affected. For example, one reasonable hypothesis may be that the effect of nutrition on type II diabetes risk is entirely mediated by BMI. However, the mediation in this instance would be through the entire BMI process. If BMI is measured only on a relatively small number of occasions, then we would expect only a proportion of the effect of nutrition on type II diabetes risk to be mediated by the observed measures of BMI.

## Summary

In this commentary we have highlighted concepts and methods from the field of causal inference, many of them recent contributions, which we believe to be relevant in life course studies. We have discussed how the language of potential outcomes can help to articulate the precise question(s) being asked and how causal DAGs—distinct from conceptual frameworks—should be carefully drawn and interrogated and missing data and measurement error mechanisms included.

## Funding

R.D. acknowledges support from a Sir Henry Dale Fellowship jointly funded by the Wellcome Trust and the Royal Society (Grant Number 107617/Z/15/Z); and B.D.S. acknowledges support from the Economic and Social Research Council (grants ES/I025561/1, ES/I025561/2 and ES/I025561/3).

**Conflict of interest:** None declared.
