# Commentary: Incorporating concepts and methods from causal inference into life course epidemiology

**DOI:** 10.1093/ije/dyw367

**Published:** 2017-03-14

**Authors:** Bianca L De Stavola, Rhian M Daniel

Published in Int. J. Epidemiol. (2016) 45 (4): 1006-1010; doi:10.1093/ije/dyw103

This commentary was originally published with errors in an equation on p.1007 in section ‘Total and joint effects’.

The equation initially was shown as 
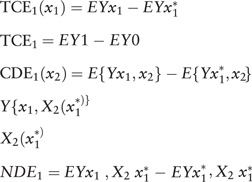


When it should have been 
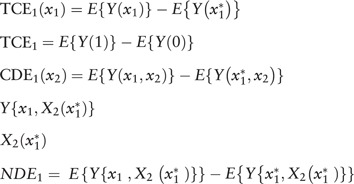


This has now been corrected. The publishers apologise for the error.

